# Weak Value Amplification Based Optical Sensor for High Throughput Real-Time Immunoassay of SARS-CoV-2 Spike Protein

**DOI:** 10.3390/bios14070332

**Published:** 2024-07-08

**Authors:** Xiaonan Zhang, Lizhong Zhang, Han Li, Yang Xu, Lingqin Meng, Gengyu Liang, Bei Wang, Le Liu, Tian Guan, Cuixia Guo, Yonghong He

**Affiliations:** 1 Shenzhen Key Laboratory for Minimal Invasive Medical Technologies, Institute of Optical Imaging and Sensing, Shenzhen International Graduate School, Tsinghua University, Shenzhen 518055, China; zxn19@mails.tsinghua.edu.cn (X.Z.); han-li22@mails.tsinghua.edu.cn (H.L.); xuyang20@mails.tsinghua.edu.cn (Y.X.); mlq21@mails.tsinghua.edu.cn (L.M.); lianggy21@mails.tsinghua.edu.cn (G.L.); wangbei22@mails.tsinghua.edu.cn (B.W.); guantian@sz.tsinghua.edu.cn (T.G.); heyh@sz.tsinghua.edu.cn (Y.H.); 2 School of Medicine, Tsinghua University, Beijing 100084, China; 3 Institute of Materials Research, Shenzhen International Graduate School, Tsinghua University, Shenzhen 518055, China; liu.le@sz.tsinghua.edu.cn; 4 School of Mechanical Engineering and Automation, Fuzhou University, Fuzhou 350108, China; 5 Jilin Fuyuan Guan Food Group Joint Research Center, Shenzhen International Graduate School, Tsinghua University, Shenzhen 518055, China

**Keywords:** weak measurement, high throughput immunoassay, optical biosensor, SARS-CoV-2 spike protein

## Abstract

The demand for accurate and efficient immunoassays calls for the development of precise, high-throughput analysis methods. This paper introduces a novel approach utilizing a weak measurement interface sensor for immunoassays, offering a solution for high throughput analysis. Weak measurement is a precise quantum measurement method that amplifies the weak value of a system in the weak interaction through appropriate pre- and post-selection states. To facilitate the simultaneous analysis of multiple samples, we have developed a chip with six flow channels capable of conducting six immunoassays concurrently. We can perform real-time immunoassay to determine the binding characteristics of spike protein and antibody through real-time analysis of the flow channel images and calculating the relative intensity. The proposed method boasts a simple structure, eliminating the need for intricate nano processes. The spike protein concentration and relative intensity curve were fitted using the Log-Log fitting regression equation, and R^2^ was 0.91. Utilizing a pre-transformation approach to account for slight variations in detection sensitivity across different flow channels, the present method achieves an impressive limit of detection(LOD) of 0.85 ng/mL for the SARS-CoV-2 the severe acute respiratory syndrome coronavirus 2 (SARS-CoV-2) spike protein, with a system standard deviation of 5.61. Furthermore, this method has been successfully verified for monitoring molecular-specific binding processes and differentiating binding capacities.

## 1. Introduction

In recent years, the severe acute respiratory syndrome coronavirus 2 (SARS-CoV-2) has emerged as a significant threat to global public health, leading to the worldwide coronavirus disease (COVID-19) pandemic [1,2]. As of 21 June 2023, the global confirmed cases of SARS-CoV-2 infection stand at 768,187,096, with a death toll of 6,945,714 [3]. Infected individuals commonly exhibit symptoms such as fever and cough [4]. Importantly, even before symptom onset, infected individuals can transmit the virus effectively [5]. The primary modes of SARS-CoV-2 transmission include droplets, aerosols, and direct contact [6,7]. Numerous measures have been implemented globally to combat SARS-CoV-2 [8,9], effectively controlling the COVID-19 pandemic. On 5 May 2023, the Director-General of the World Health Organization officially declared an end to a public health emergency of international concern triggered by COVID-19 [10]. However, SARS-CoV-2 persists and threatens human health, making its immunoassay paramount.

The spike protein is a structural protein present on the surface of the SARS-CoV-2 virus. It comprises two subunits, S1 and S2, with the receptor-binding domain (RBD) of the S1 subunit interacting with the human angiotensin-converting enzyme II(ACE2) receptor, while the S2 subunit facilitates the fusion of the viral membrane with the host cell membrane, resulting in the transfer of viral genetic material into human cells and subsequent infection [11,12]. Neutralizing antibodies are a promising therapy for inhibiting SARS-CoV-2 infection in the human body [13,14,15]. These antibodies bind to the spike protein, blocking its interaction with the ACE2 receptor and reducing the virus’s infectivity [16,17]. Therefore, studying the binding properties between spike proteins and neutralizing antibodies is crucial. Such investigations can aid in developing and screening potent neutralizing antibodies that effectively inhibit SARS-CoV-2 infection. Overall, these efforts can contribute to the prevention and treatment of COVID-19.

Optical biosensors exhibit excellent performance in immunoassay [18]. This global epidemic shows that large-scale application, high-precision immunoassay methods have significant implications for public health safety. The label-free optical immunoassay methods using optical biosensors based on various principles such as surface-enhanced Raman scattering (SERS), total internal reflection (TIR) [19], bio-layer interferometry (BLI) [20], and surface plasmon resonance (SPR) [21,22,23,24]. These optical biosensors find extensive applications owing to their remarkable sensitivity and immunity to interference. In 1988, the weak value amplification effect was discovered by Aharonov, Albert, and Vaidman [25]. Weak measurement is a precision measurement method that generates the weak value amplification effect under weak interaction. Since the turn of the century, weak value amplification techniques have progressively found utility in precision measurement [26,27,28]. In line with this trend, our research group has been actively investigating the application of weak measurement techniques in label-free biosensing since 2015, yielding remarkable outcomes [29,30,31].

This study presents a novel optical approach for conducting high-throughput immunoassays targeting viral Spike proteins. Our research builds upon the optical weak measurement interface-based sensor previously proposed by our team, enabling real-time monitoring of the immune process across a single, large area [31]. Using SARS-CoV-2 as a case study, we successfully monitored the binding process between Spike proteins and antibodies at various concentrations, achieving a limit of detection(LOD) of 0.85 ng/mL.

Our research yielded outstanding results for binding a single Spike protein to an antibody and enabled simultaneous monitoring of the binding process involving multiple mutant Spike proteins and antibodies. Moreover, this sensing method boasts a simple structure, high robustness, low resource consumption, and broad applicability. It holds great potential for integration with microfluidic systems and other biochip technologies.

## 2. Materials and Methods

### 2.1. Materials

Phosphate buffered solution (PBS, powder) and normal human serum were purchased from Solarbio Science&Technology Company (Beijing, China). PBS at pH 7.3 with a concentration of 0.01 mol/L provides an environment for biomolecular interactions. Dopamine hydrochloride and Tris (hydroxymethyl) aminomethane were provided by Aladdin (Shanghai, China). Prepare a solution of dopamine hydrochloride at a concentration of 1 mg/mL dissolved in Tris solution at a concentration of 0.01 mol/L and store it away from light. Recombinant SARS-CoV-2 Spike Protein RBD-SD1, Recombinant SARS-CoV-2 S Protein RBD (XBB.1.5, C-6His), Recombinant SARS-CoV-2 S Protein RBD (Omicron, B.1.1.529, C-6His), Recombinant 2019-nCoV S Protein RBD (B.1.617.2, C-6His) and Anti-2019-nCoV S1 mAb (5D9) were purchased from Suzhou Novoprotein Scientific Co., Ltd. (Suzhou, China). No protein-blocking solution (2%) was purchased from Sangon Biotech (Shanghai, China).

The materials employed in the optical sensing component consisted of a ZF6 glass prism obtained from Alpha Optics Co., Ltd. (Fuzhou, China), and a resin-based chip fabricated via 3D printing.

### 2.2. Methods

**Figure 1:**
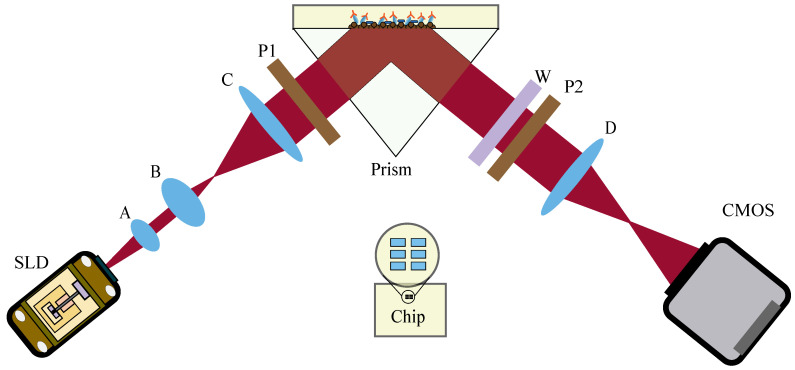
This is the weak measurement-based interfacial sensing structure. A is the collimated lens, and lens B and C construct the beam-spreading structure. P1 and P2 are polarizers, and W is a quarter-wave plate. D is the imaging lens. The insert image shows the flow channel layout of the chip.

The system architecture is presented in Figure 1, illustrating the overall structure. Initially, the SLD (superluminescent diode) (5 mW, IPSDD0804, Inphenix, Inc., Livermore, CA, USA) light source emits light through an optical fiber. Subsequently, the light is collimated by lens A (f = 30 mm, #22-487, Edmund Optics Inc., Barrington, NJ, USA), and its beam area is amplified using a beam-spreading structure (lens B [f = 50 mm, GCL-010652BF, Daheng Optics, Beijing, China] and lens C [f = 150 mm, GCL-010605BF, Daheng Optics, Beijing, China]). To ensure spatial filter uniformity, a 500 μm diameter pinhole (P500HW, Thorlabs Optronics [Shanghai], Shanghai, China)) is employed as a spatial filter at the confocal point of the beam spreading structure. The collimated beam, with a diameter of approximately 50 mm, enters the weak measurement sensing structure.

The weak measurement sensing structure consists of a pre-selection, coupling prism, quarter-wave plate (GCL-060802, Daheng Optics, Beijing, China), optical rotator, and post-selection. Each pre-selection and post-selection incorporates a polarizer (LPVIS100-MP2, Thorlabs Optronics [Shanghai], Shanghai, China). The polarization axis of the pre-selected state is inclined at an angle of π/4 relative to the vertical direction. The light passing through the pre-selected state is coupled to the chip by a prism, utilizing an angle of incidence larger than the critical angle for total internal reflection. Consequently, the interactions between biomolecules and the detection interface induce a phase difference between the p-polarized and s-polarized components due to changes in the surface refractive index. This phase difference is further amplified by the weak measurement structure. Finally, an imaging lens (f = 100 mm, GCL-010615, Daheng Optics, Beijing, China) images the detection light onto a CMOS (Complementary Metal Oxide Semiconductor) (ASI533MMPro, Suzhou ZWO, Suzhou, China).

**Figure 2:**
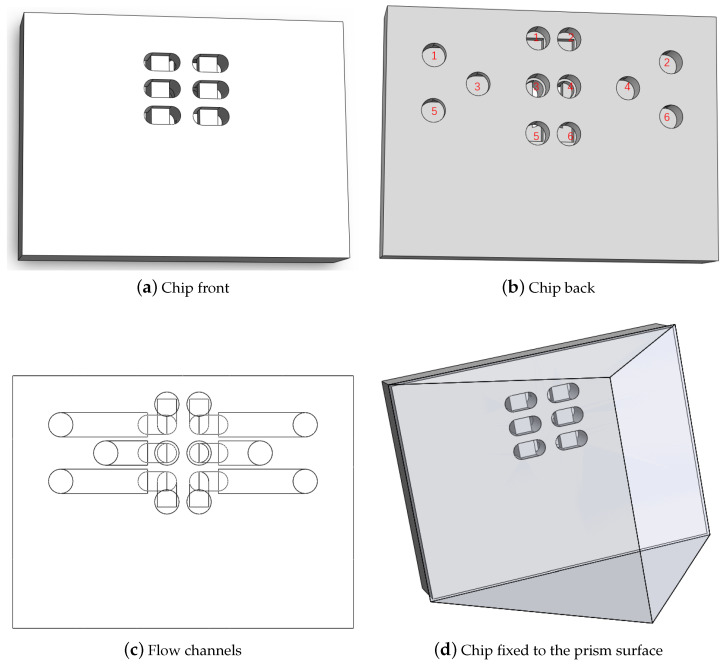
This is the chip structure. (**a**) The six grooves of the chip. (**b**) Chip grooves have corresponding inlets and outlets labeled with the same number. (**c**) The internal structure of flow channels. (**d**) The chip and the prism form flow channels flowing through the surface of the prism.

The flow channel system is comprised of a chip and a syringe pump. The chip is designed with six grooves, each containing inlets and outlets at both ends, as illustrated in Figure 2. The grooves are bonded to the prism surface using adhesive, forming the flow channel. This flow channel facilitates the flow of the solution through the prism surface. To initiate the flow of the solution, one end of the flow channel is connected to the solution while the other is connected to the syringe pump. In this way, the syringe pump allows the solution to flow through the prism surface by extracting the solution. This chip enabled simultaneous experiments with five concentration gradients and provided a reference channel. The design of the reference channel effectively suppressed noise and temperature drift, which has been thoroughly investigated in our previous work [31]. In this study, we acknowledged that inconsistencies in sensitivity among the final detection channels could arise from non-uniformity in the light source and suboptimal optics when imaging more extensive interfaces. To address this concern, we performed sensitivity calibration and normalization for each detection channel before conducting formal experiments. This calibration involved utilizing sodium chloride to alter the refractive index of the PBS buffer.

The experimental procedure can be divided into two main phases: assay surface functionalization and specific binding. During the surface functionalization process, we utilized 0.01 M Tris solution with a concentration of 1 g/L dopamine. We allowed it to flow over the prism surface for 20 min to form a polydopamine layer with excellent adhesion. Subsequently, the solution to be measured was passed through this layer, leading to the nonspecific adherence of the spike protein to the polydopamine surface. Then, we complete the closure of the polydopamine layer using a 2% no-protein blocking solution.

After 50 min of pass-through buffer, a baseline was conducted. To initiate the specific binding process, antibodies were passed through the chip at the same rate as the buffer, ensuring a constant velocity and maintaining relatively stable pressure inside and outside the channel throughout the binding process. This uniform speed is crucial as pressure fluctuations can significantly affect the refractive index of the glass, which directly correlates with the biological process. By maintaining a constant velocity, we minimized the impact of pressure on the experimental results.

For the experimental groups, we designed five spike protein gradients of 0.625, 1.25, 2.5, 5, and 10 μg/mL as experimental groups. Also, we designed an inactive channel without fixed antigen and passed only the buffer as a reference in the specific phase. This experimental setup was realized using the six-channel chip, as depicted in Figure 2. Image processing using MATLAB (R2019a).

## 3. Results

### 3.1. High-Throughput Immunoassay

**Figure 3:**
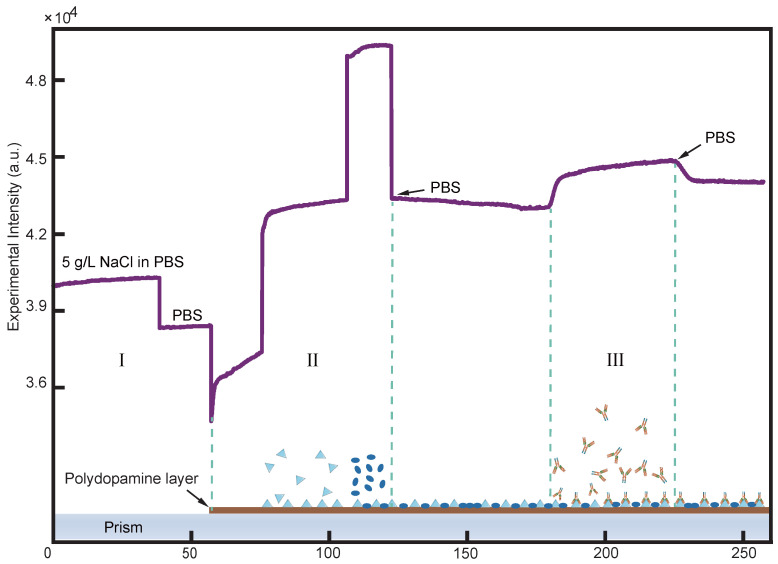
This is an entire experimental process for the detection of flow channels, starting with the first stage I, where the refractive index change is artificially introduced by changing the NaCl solution (5 g/L) dissolved in PBS solution (0.01 mol/L), thus completing the normalization of the sensitivity of the different detection sites on the detection surface; the second stage II is the stage of surface functionalization, where the solutions passed in turn are dopamine Tris solution (1 mg/mL), spike protein (0.625 μg/mL), and no-protein closure solution (2%); the third stage III is the passage of PBS to form a baseline after which the antibody solution (5 μg/mL) is passed in for specific binding, and finally changed back to PBS to read out the response value. The bottom of the figure shows the whole process of molecular binding in the experiment, where the triangle, oval and Y-shaped graphs represent the spike protein, the polymer (in the no protein blocking solution) and the antibody, respectively.

In the embodiment of the fundamental performance of the sensing method, we have employed the spike protein of the SARS-CoV-2 virus as the target antigen for detection to achieve real-time monitoring through multiple concentration gradients.The entire experimental process is shown in the Figure 3.

**Figure 4:**
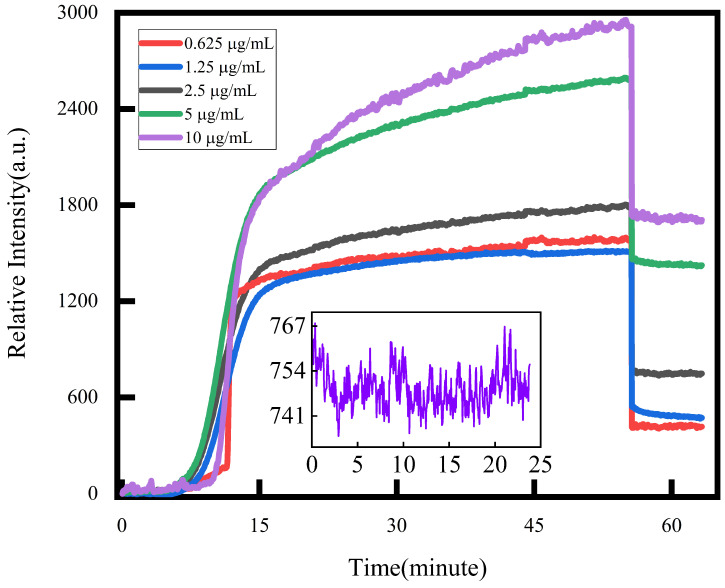
This is the binding process of different concentrations of spike protein. The insert image is the light intensity change of PBS flowing through the channel.

It is evident from the result Figure 4 that the results from the specificity phase demonstrate a positive correlation between the concentration of the spike protein being tested and the magnitude of light intensity change. This change varies from low to high across the five experimental groups. During the transition from the specific binding phase to the final PBS buffer phase, the change curve exhibits a noticeable decline in light intensity. This decline can be attributed to the body effect, which arises from the disparity in refractive indices between the experimental solution and the final stage of the PBS buffer. Consequently, this discrepancy slightly alters the sensor’s response value following solution changes. We replicated the experiment illustrated in Figure 4 three times and computed the relative intensities that signify the degree of binding, as illustrated in Figure S1 (refer to Supplementary Information).

To ascertain the response value for the entire binding process, evaluating the variation between the PBS buffer before and after the experimental solution is necessary. The curve within the experimental section effectively portrays the progression of specific binding. We employed PBS buffer for 20 min in our sensor. Through the implementation of our novel approach, we achieved a reduced standard deviation $\delta_{S}=5.61$, from the insert image of Figure 4.

We used the Log-Log fitting regression equation [32] to fit the standard curve of spike protein RBD concentration and relative intensity, as shown in Formula (1).

(1) lg(y)=a×lg(x)+b

**Figure 5:**
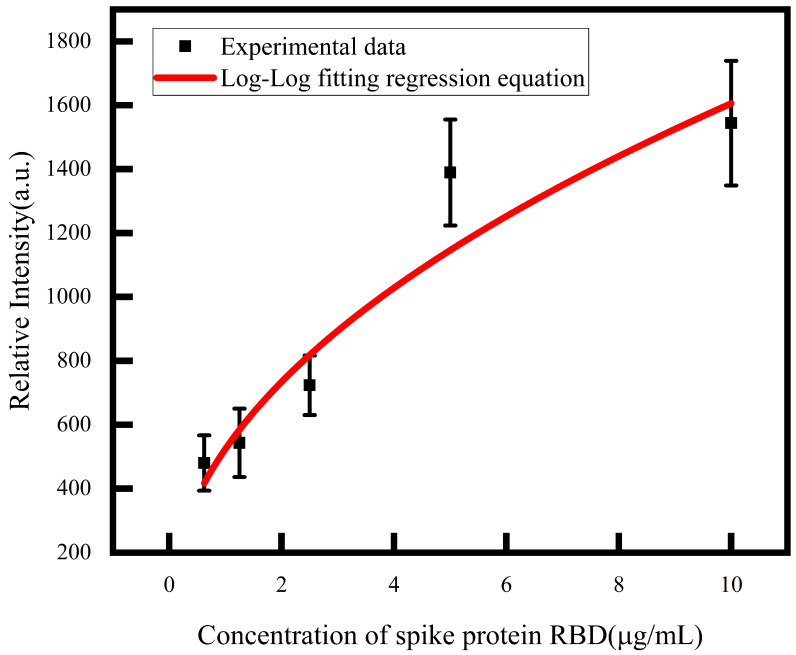
Fitting curve of relation between spike protein RBD concentration and relative intensity (The error bar represents the standard deviation of the experiment repeated three times).

The standard curve obtained by fitting is shown in the Figure 5, and R^2^ is 0.91. Therefore, the Log-Log fitting regression equation can fit the standard curve of spike protein RBD concentration and light intensity well.

According to the function shown in Formula (1), we take three times the standard deviation (5.61) as the *y* value and find the corresponding *x* at this time, which is the lowest detection limit. Therefore, the LOD is 0.85 ng/mL.

**Figure 6:**
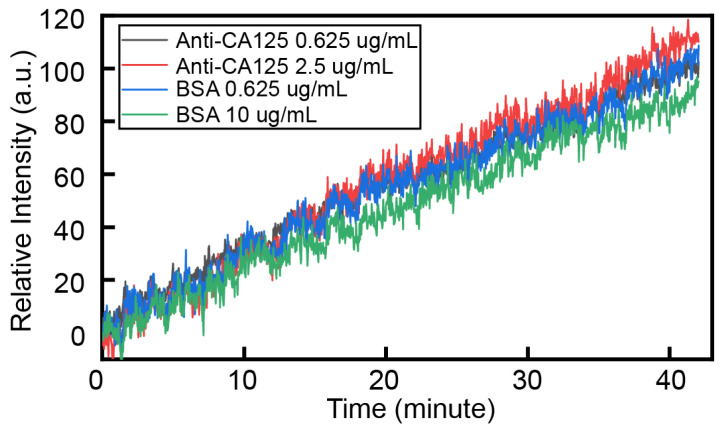
This contains the response curves for the process of molecule passing through the sensing surface. The molecules are rabbit anti-CA125 at concentrations of 0.625 μg/mL and 2.5 μg/mL and BSA at concentrations of 0.625 μg/mL and 10 μg/mL.

Furthermore, considering the actual assay, we cannot know whether the components in the sample will undergo non-specific binding. Therefore, as shown in Figure 6, two concentrations, i.e., anti-CA125 0.625 μg/mL, 2.5 μg/mL, and BSA 0.625 μg/mL and 10 μg/mL, were added in this work as non-specific validation targets for this sensor scheme, as the spike protein’s concentration remained at 10 μg/mL. As can be seen in Figure 6, the change in relative light intensity caused by non-specific binding is only about 100, much smaller than the relative light intensity caused by several concentrations of spike proteins demonstrated in Figure 4. This indicates that the amount of non-specific binding is small in practical applications.

In practical applications, in order to avoid non-specific effects, we set the relative intensity caused by non-specific binding as 150, so we think that the relative intensity above 150 is caused by specific binding. According to the fitting model, we set *y* to 150, and then the corresponding *x* is the lowest detectable spike protein RBD concentration, so our detection range is not lower than this concentration, which is 76.4 ng/mL.

### 3.2. Characterization of the Binding of Spike Protein of Mutants to Antibody

**Figure 7:**
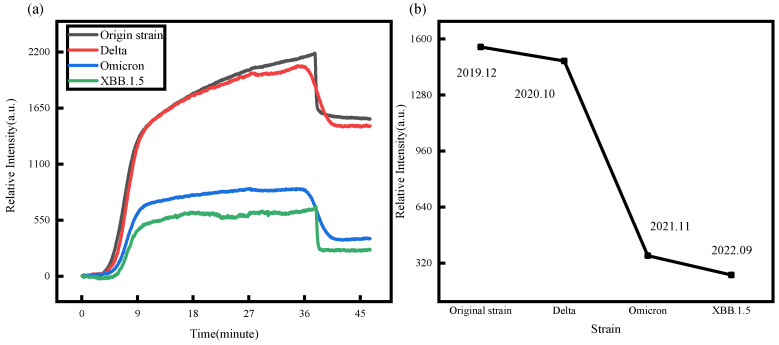
(**a**) This is the process by which the mutant spike protein binds to the antibody. (**b**) The binding amount of RBD and antibody of mutants(the time when the SARS-CoV-2 and variants were first discovered is labeled in the figure).

Given the continuous mutation of the virus, it is essential to ensure the sensor’s capability in detecting mutant strains, although this heavily relies on the specific recognition process facilitated by the antibody. While the RBD segment of the spike protein exhibits sufficient specificity towards the antibody, the increasing number of mutation sites results in altered affinity between the mutated spike protein and the original monoclonal antibody. Therefore, our study selected four viral strains, Original strain, Delta (B.1.617.2), Omicron (B.1.1.529), and XBB.1.5, each with a concentration of 5 μg/mL, as targets for the assay. Repeating the modification and specific binding steps described in Section 3.1, we obtained compelling outcomes, presented in Figure 7a. These results robustly demonstrate the real-time monitoring ability of our sensor in capturing the kinetic process of specific binding between viral spike proteins and antibodies. Notably, Figure 7b allows us to discern the affinity of the original strain monoclonal antibody towards various mutant strain spike proteins. The affinity ranking is as follows: Original strain > Delta > Omicron > XBB.1.5. As the SARS-CoV-2 continues to mutate, there has been a gradual decrease in the binding amount between the spike protein RBD and Anti-2019-nCoV S1 mAb (5D9) of the mutant strain. This finding suggests that the binding sites of the spike protein may have changed.

## 4. Discussion

For the fundamental performance analysis of the sensor, a clear differentiation in response among different concentrations is observed in the single gradient experiment, as depicted in Figure 4. Notably, the system demonstrates exceptional detection limits of 0.85 ng/mL.

We compared the ideal detection limit of several commercial kits and optical biosensors for the detection of SARS-CoV-2, as shown in Table 1.

Commercial kits are convenient to use but do not provide quantitative results. In contrast, our weak value amplification-based optical biosensor is able to offer competitive LOD compared to other biosensors, as demonstrated in Table 1.

As can be seen, the optical system only reflects the biophysical processes occurring at the detection interface, and there is no point in discussing the specificity of the physical system itself. The main factor limiting the detection limit in practical use is the non-specificity due to the biochemical sensing scheme rather than the optical system itself. Therefore, choosing a less non-specific molecular binding scheme is an effective route to improving the performance of the sensing system, e.g., metal nanoparticles modifying the antibody for detection, and amino silanes functionalized and modifying the antibody using an amino silane at the glass interface of the assay are schemes that can effectively reduce the non-specific leakage introduced by the use of polydopamine membrane adhesion-closure. Meanwhile, in terms of experimental protocol design, a flow channel introducing a known irrelevant protein molecule instead of the blank buffer as a reference channel can be chosen to eliminate the non-specific effects, the non-specific effects of which may need to be further explored in future research work.

Furthermore, several factors contribute to inter-group variability. The first factor involves the mechanical fixation of coupling prisms with chips across different experimental groups. This fixation introduces slight deviations in the discrete optical systems, potentially impacting the prism angle, position, and, consequently, the channel location difference at the detection site between experimental groups. As a result, an additional phase response is introduced, which is unavoidable under laboratory conditions. However, this issue can likely be addressed in industrial prototypes by integrating high-precision mechanical parts.

Secondly, variations in the overall experimental environment exist between the different groups. These variations encompass fluctuations in light intensity emitted by the light source, noise caused by scattered particles, and dark current noise in the CMOS detector. Additionally, mechanical errors in the rotation angle of the polarizer and waveplate can induce phase variations and subsequently affect the intensity values of the response light.

It is worth mentioning that the utilization of a 27 mm × 20 mm equilateral prism reflecting surface in this approach, alongside the overall dimensions of the six flow channels measuring approximately 5 mm × 3.5 mm, enhances the feasibility of integrating this method into microfluidic chips utilizing 3D printing, PDMS and any other foundational technologies.

In the comparative binding capacity experiments Figure 7, our findings indicate that the original strain of antibody exhibits a relatively strong binding capacity to both the original strain and Delta variant. However, we observed a significant decrease in binding capacity to Omicron and XBB.1.5, which aligns with findings from other studies [38,39,40].

## 5. Conclusions

In conclusion, this study has introduced a novel high-throughput immunoassay method utilizing a weak measurement interface sensor. The achieved concentration resolution for the SARS-CoV-2 spike protein was an impressive LOD of 0.85 ng/mL. Furthermore, the experiment successfully demonstrated the discernible variance in detection and binding capacity of the molecular-specific binding process when applied to different variants of the virus’s spike protein.

This approach offers a more fundamental platform with remarkable scalability compared to alternative sensing methods. For instance, surface sensitization methods present increased adsorption sites, while nanoparticle-modified antibodies contribute to extended response values. Employing surface and biomolecular chemistry approaches makes it feasible to further enhance the assay’s sensitivity. Integrating microfluidic technologies opens up possibilities for expanding the number of parallel channels, enabling even higher throughput.

The simplicity of the sensing methods described in this paper ensures reliability and establishes a robust foundation for immunoassays. The findings of this research contribute to the development of advanced immunoassay techniques in the field, offering a valuable tool for various applications in healthcare and diagnostics. Future investigations may explore additional optimizations and the potential for broader implementation of this approach.

## Figures and Tables

**Table 1:** The detection limit of several common commercial kits and optical biosensors.

Method	Analyte	LOD	Ref.
BinaxNOW COVID-19 Antigen Self-Test	Nucleocapsid protein	140.6 TCID50/mL	[33]
2019-nCoV Antigen Detection Kit (Colloidal gold method)	Nucleocapsid protein	600 TCID50/mL	[34]
Colorimetric biosensor	Spike protein	11 ng/mL	[35]
Electrochemical Immunosensor	Spike protein	299.30 ng/mL (for ACE2), 38.99 ng/mL (for CD147)	[36]
Surface Plasmon Resonance biosensor	Spike protein	8.34 ng/mL	[37]
This work	Spike protein	0.85 ng/mL	

## Data Availability

The data presented in this study are available on request from the corresponding author.

## Supplementary Materials

The following supporting information can be downloaded at: https://www.mdpi.com/article/10.3390/bios14070332/s1, Figure S1: This is the relative intensity induced by varying concentrations of spike protein (The error bar represents the standard deviation of the experiment repeated three times).

## Abbreviations

The following abbreviations are used in this manuscript:

RBD	Receptor-binding domain
ACE2	Angiotensin-converting enzyme II
SERS	Surface-enhanced Raman Scattering
BLI	Bio-Layer Interferometry
SPR	Surface plasmon resonance
LOD	Limit of detection
PBS	Phosphate buffered solution
SLD	Superluminescent diode
CMOS	Complementary Metal Oxide Semiconductor
