# Supplementary material for: Weak Value Amplification Based Optical Sensor for High Throughput Real-Time Immunoassay of SARS-CoV-2 Spike Protein
Source: Biosensors (Basel). 2024 Jul 8;14(7):332. doi: 10.3390/bios14070332 (PMC11274545; doi:10.3390/bios14070332)
Supplement: Supplementary file 1 [file biosensors-14-00332-s001.zip › biosensors-3002643-supplementary.pdf]

Supplementary Information

1. The amount of SAR-CoV-2 spike protein binding

The SARS-CoV-2 spike protein was specifically bound to the antibody at varying concentrations, ranging from 0.625 to 10 μg/mL. The findings from the experiment measuring the amount of the SARS-CoV-2 spike protein binding are illustrated in Figure S1.

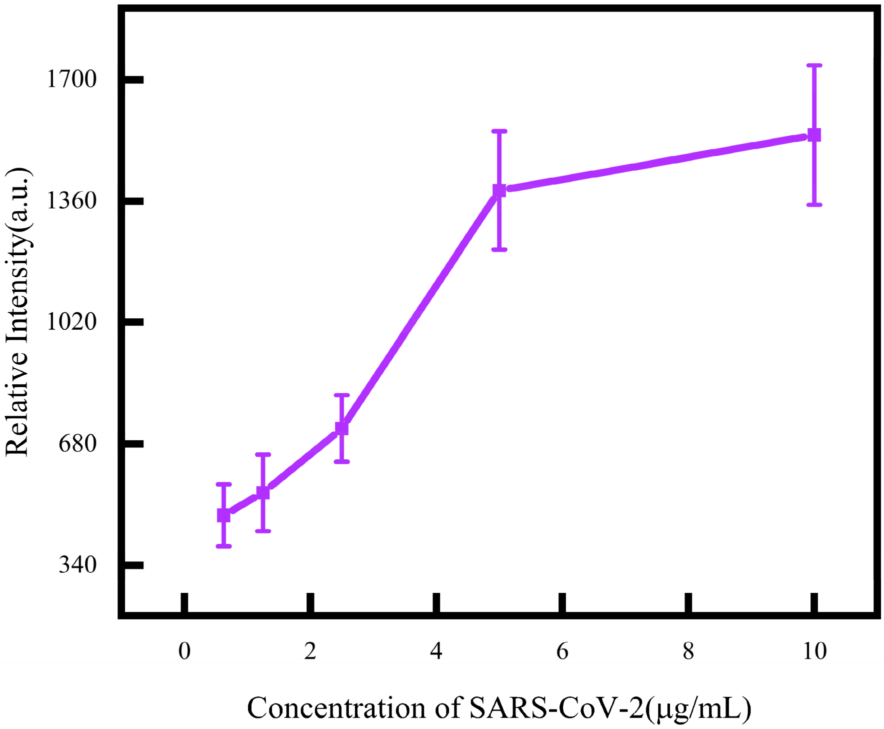

Figure S1. This is the relative intensity induced by varying concentrations of spike protein(The error bar represents the standard deviation of the experiment repeated three times).
